# A Confinement with Rupture of the Uterus

**Published:** 1881-02

**Authors:** P. W. Van Peyma

**Affiliations:** Custrin


### A CONFINEMENT WITH RUPTURE OF THE UTERUS. —REPORTED BY DR. NIEPRASCH, OF CUSTRIN.

FROM THE GERMAN BY P. W. VAN PEYMA, M. D.

On the evening of the 28th of October, 1879, I was called to the village of Alt Drewitz, near Cüstrin, to see Mrs. P., in labor for the seventh time. The general development, including that of the pelvis, was normal. In consequence of malposition of the child, she had on three former occasions required medical aid. The husband informed me that the present child had presented by the feet, and was already completely delivered, but that the midwife did not venture to tie the cord, from the fact of its being a case of monstrosity. Arriving at the bed of the patient, a large pool of blood was noticed upon the floor, which, in consequence of a very great hæmorrhage, had soaked through the mattress and collected on the floor beneath. The effects of this serious hæmorrhage were noticeable at the first glance, as also upon further examination of the patient. Her countenance showed the well known anæmia, and was covered with perspiration; the pupil dilated; the respiration infrequent and deep; the body cold; the radial artery could not be felt, and the cardiac impulse was retarded and scarcely audible. Upon removal of the covering there was found, between the thighs of the patient, a newly-born child, whose head was apparently a raw unformed mass. Upon closer examination, this proved to be the uterus enclosing the head of the child, and seven or eight centimetres removed from the vulva. The uterus was still attached to the body by means of the round ligament, and slight depressions upon its surface indicated the former attachment of the ligaments. Between the uterus and the vulva were loops of the small intestine.

It was evident that the midwife in her endeavor to reach the arms of the partially delivered child, had pressed through and torn the recto-vaginal septum. In her further manipulations, the uterus, robbed of its support, had been drawn down. Mistaking this for a deformed head, and seeking by the employment of force to end the labor, the ligaments were ruptured, the bowels pulled through, and the picture detailed presents itself.

About two hours after the completion of these events, I arrived at the scene of misfortune; the fully developed child had of course died, and nothing remained but to remove the head and placenta from the cavity of the uterus. After considerable exertion this was accomplished without resource to the knife. Considering the patient as lost, I was the more inclined to forego all further operative interference on account of the condition of the patient. Any additional shock would most certainly have resulted in immediate death. I satisfied myself with simply inwrapping the intestines and other presenting parts in oiled linen, and enjoined the husband to call on me the next morning in the event of his wife still being alive. Early the next morning the man presented himself, and informed me that his wife had revived, was able to speak, &c.

I resolved, by section of the round ligament, to sever the connection between the body and uterus. Dr. Gerlach, whose assistance I requested, kindly accompanied me. Upon removal of the oil cloth a strong odor of putrefaction was noticable; the uterus presented itself as a dark brown steaming mass. The extruded intestine was so strongly injected and greatly swollen, that Dr. G. at first believed it to be the large intestine. The general condition of the patient was that of the deepest depression, the body was still cold, the pulse 200 and small, the mental faculties clear.

The removal of the uterus was, of course, easily accomplished, by simple ligation and section of the round ligament. The intestine still remained to be replaced and, fortunately, upon this being done, it was found that the bladder, which was distended nearly closed the opening, acting as a valve. Later a large sponge, saturated with a 2 per cent. carbolic acid solution was passed into the vagina, the bladder being evacuated by means of a catheter.

The subsequent treatment of this very sick patient was pursued under the most disadvantageous sanitary surroundings. The apartments consisted of two small rooms, warmed by a single stove, and occupied by seven persons. The bed was placed in the front room. The husband acted as nurse and cook, and was also instructed in the use of the catheter. A neighboring woman occasionally lent a little aid in the general care. On account of the distance, my visits at the most could be rendered only once a day.

In the treatment all antiphlogistics were set aside, a glass of red wine given in teaspoonful doses during the twenty-four hours, small quantities of broths, etc., frequently repeated. To allay in some measure the great thirst, small pieces of ice were administered; with a view to check the movements of the bowels, opium and lig. chlori were given in mint water.

Oct. 30th a high reactive fever, not preceded by a chill; pulse small and so frequent as to be with difficulty counted; temp. 42° C; thirst very great and ice in great demand; the sponge was daily removed and replaced by a clean one; the discharge was great in amount and fetid in odor. The patient lay in this discharge mixed with urine, and as the bed was thoroughly soaked, carbolic acid was simply added, and a clean blanket spread over it. In spite of the inclemency of the weather, a window remained constantly open.

Oct. 31. After a sleepless night, the symptoms remained unchanged; no action of bowels; great tympanites, tenderness over pubic region, due to the general condition, was not marked.

The patient had now control of the bladder; mind remained perfectly clear. The husband informed me that the intestines had again fallen down, but upon examination he was found to be mistaken, the presenting tumor being the recto-vaginal septum, which was immediately replaced by means of the sponge, and afterwards remained in position.

Nov. 2. First movement of the bowels; other symptoms remaining the same. The stool was principally mucous, but contained small masses of hardened fecal matter. For the next four days there were from four to eight motions a day. During this time the abdominal distention and tympanites disappeared. The abdomen was but slightly sensitive; the pulse gradually dropped to 140; the thirst became less; and a slight appetite established itself. The sanitary conditions remained much the same.

Nov. 7. Condition much the same; the patient takes acid muriatic, and occasionally potassa nitrat.

Nov. 8. The action of the bowels stopped until the 12th, when ol ricini, grams 15, reinforced by an injection, resulted in a free evacuation of a soft consistency. From this time on, the discharge lost its fetid character, was much diminished in quantity, and on Nov. 16 had assumed the character of laudable pus.

The internal parts had by this time healed, and the pus had its origin in the vaginal septa. By pressure over the pubic region two hardened bands can be felt extending to either side, and supposed to be the indurated broad ligaments.

Although the appetite was now good, and the strength improved, the pulse still continued at 140 until Nov. 26, when the pus formation ceased, and with it the fever.

Small sponges were introduced three times daily, a nourishing diet, and an occasional dose of Ol. Ricini ordered.

At the present time (July, 1880) the finger, after passing up about eight centimetres, feels the roof-shaped vaginal septum, closed above in consequence of the inflammation. An examination with the speculum has not been made as yet in consideration of the weakness of the parts and the general condition of the patient. The ovaries can be felt as firm and hardened bodies.

Some twenty years ago Prof. Crede related to me a similar case.

As medical literature, so far as I am aware, does not include a similar case, I have considered the case worth recording, both on accouut of its rarity and the favorable result. At the present time the woman has a healthy, rosy complexion, and attends to her household duties with remarkable ease and vigor.—*Berliner Klinische Wochenschrift.*

